# Reperfusion‐induced sustained ventricular tachycardia, leading to ventricular fibrillation, in chronically instrumented, intact, conscious mice

**DOI:** 10.14814/phy2.12057

**Published:** 2014-06-27

**Authors:** Heidi L. Lujan, Stephen E. DiCarlo

**Affiliations:** 1 Department of Physiology, Wayne State University School of Medicine, Detroit, 48201, Michigan

**Keywords:** arrhythmia, murine model, reperfusion injury

## Abstract

Reperfusion‐induced lethal ventricular arrhythmias are observed during relief of coronary artery spasm, with unstable angina, exercise‐induced ischemia, and silent ischemia. Accordingly, significant efforts are underway to understand the mechanisms responsible for reperfusion‐induced lethal arrhythmias and mice have become increasingly important in these efforts. However, although reperfusion‐induced sustained ventricular tachycardia leading to ventricular fibrillation (VF) has been recorded in many models, reports in mice are sparse and of limited success. Importantly, none of these studies were conducted in intact, conscious mice. Accordingly, a chronically instrumented, intact, conscious murine model of reperfusion‐induced lethal arrhythmias has the potential to be of major importance for advancing the concepts and methods that drive cardiovascular therapies. Therefore, we describe, for the first time, the use of an intact, conscious, murine model of reperfusion‐induced lethal arrhythmias. Male mice (*n* = 9) were instrumented to record cardiac output and the electrocardiogram. In addition, a snare was placed around the left main coronary artery. Following recovery, the susceptibility to sustained ventricular tachycardia produced by 3 min of occlusion and reperfusion of the left main coronary artery was determined in conscious mice by pulling on the snare. Reperfusion culminated in sustained ventricular tachycardia, leading to VF, in all nine conscious mice. The procedures conducted in conscious C57BL/6J mice, a strain commonly used in transgenic studies, can be utilized in genetically modified models to enhance our understanding of single gene defects on reperfusion‐induced lethal ventricular arrhythmias in intact, conscious, and complex animals.

## Abstract (precis)

We describe, for the first time, the use of an intact, conscious, murine model of reperfusion‐induced lethal arrhythmias. This model has the potential to be of major importance for advancing the concepts and methods that drive antiarrhythmic therapies.

## Introduction

Sudden cardiac death, due to coronary artery occlusion, accounts for 300,000–400,000 deaths annually in the United States (Myerburg and Castellanos 1997). Reperfusion, following coronary artery occlusion, may account for some cases of sudden cardiac death (Manning and Hearse 1984; Van Wagoner and Bond 2001). Specifically, reperfusion after a brief period of myocardial ischemia can lead to lethal arrhythmias (Tennant and Wiggers 1935; Wit and Janse 2001). In this context, Prinzmetal et al. (1959) first described a variant form of resting, ST segment elevation angina caused by coronary artery spasm. Importantly, reperfusion, after seconds or minutes of coronary artery spasm‐induced ischemia, can induce lethal ventricular arrhythmias (Leary 1934; Prchkov et al. 1974; Maseri et al. 1978, 1982; Kerin et al. 1979; Myerburg et al. 1992; Sanna et al. 2009). Thus, although little tissue damage occurs during the ischemia, life threatening arrhythmias occur during reperfusion.

Accordingly, significant efforts are underway to develop safe and effective antiarrhythmic therapies. This requires an understanding of the mechanisms responsible for reperfusion‐induced lethal arrhythmias and mice have become increasingly important in these efforts. However, multiple systems and regulatory strategies interact to control reperfusion‐induced lethal arrhythmias (Wit and Janse 2001). Accordingly, studies investigating reperfusion‐induced lethal arrhythmias must be conducted in complex models with multiple systems and regulatory strategies to fully appreciate the physiological context. Currently, these investigations are mainly performed under conditions remote from the normal in vivo condition; thus, the extrapolation to the in vivo situation and the facilitation of translational aspect of the findings are limited (Stables and Curtis 2009).

Accordingly, a chronically instrumented, intact, conscious murine model of reperfusion‐induced lethal arrhythmias has the potential to be of major importance for advancing the concepts and methods that drive antiarrhythmic therapies. Therefore, we describe, for the first time, the use of an intact, conscious, murine model of reperfusion‐induced lethal arrhythmias.

Specifically, chronically instrumented, intact conscious mice were instrumented to record cardiac output and the electrocardiogram (ECG) (Lujan and DiCarlo 2013, 2014). In addition, a snare was placed around the left main coronary artery (Lujan et al. 2012a; Lujan and DiCarlo 2013). Following recovery, the susceptibility to sustained ventricular tachycardia produced by 3 min of occlusion and reperfusion of the left main coronary artery was determined in conscious mice by pulling on the snare. The methodology allows for the induction of lethal arrhythmias in complex, conscious mice and may be adopted for advancing the concepts and ideas that drive antiarrhythmic research.

## Materials and Methods

### Animals

Experimental procedures and protocols were reviewed and approved by the Animal Care and Use Committee of Wayne State University and complied with The American Physiological Society's Guiding Principles in the Care and Use of Animals. The procedures were initiated in nine male C57BL/6 mice (3–4 months of age, 29.1 ± 0.88 g before instrumentation and 33.4 ± 0.74 g after completion of the studies, *P* = 0.001). No mice died during the surgical instrumentation or experimental protocols.

All surgical procedures were performed using aseptic surgical techniques. Mice were anesthetized with pentobarbital sodium (60 mg/kg i.p.), atropinized (0.05 mg/kg i.p.), intubated, and prepared for aseptic surgery. Supplemental doses of pentobarbital sodium (10–20 mg/kg i.p.) were administered if the mice regained the blink reflex or responded during the surgical procedures.

### Thoracotomy procedures

The hearts were approached via a left thoracotomy through the second intercostal space as recently described (Lujan et al. 2012a,b; Lujan and DiCarlo 2013, 2014). Subsequently, the sleeve of the pericardium that extends onto the ascending aorta was dissected free of surrounding tissue and a 1.6‐mm silicone‐type Doppler ultrasonic flow probe (Iowa Doppler Products, Iowa City, IA) was positioned around the ascending aorta as recently described (Lujan and DiCarlo 2013, 2014). The flow probe wires were tunneled subcutaneously and exteriorized at the back of the neck. Subsequently, a coronary artery occluder was passed around the left main coronary artery as recently described (Lujan et al. 2012a; Lujan and DiCarlo 2013). The two ends of the occluder were passed through guide tubing fashioned from a “modified” mouse thoracic carotid artery catheter (Braintree Scientific, Braintree, MA) and the guide tubing with the two ends of the occluder were exteriorized at the back of the neck, filled with a sterile petrolatum ophthalmic ointment (Puralube), and sealed with a stainless steel pin to prevent a pneumothorax (Lujan et al. 2012a; Lujan and DiCarlo 2013). The local anesthetic, bupivacaine, was injected (s.c.) at the incision site. Subsequently, ECG electrodes (DataSciences International, St. Paul, MN, Standard Lead Coupler Kit: 276‐0031‐001) were sutured subcutaneously in a modified lead II configuration, tunneled subcutaneously and exteriorized at the back of the neck as previously described in mice (Lujan et al. 2012a; Lujan and DiCarlo 2013, 2014).

All animals remained on the feedback‐based temperature control system and ventilator until fully recovered from the anesthesia. Once the animals regained consciousness, they were placed in a “rodent recovery cage” (Thermocare^®^ Intensive Care Unit, Braintree Scientific). Animals were returned to the housing room when fully recovered from the anesthesia and gained the ability to maintain body temperature. The nonsteroidal anti‐inflammatory agent ketoprofen (5 mg/kg s.c.) and the antibiotic cefazolin (10 mg/kg s.c.) were continued for 2 days. At least 10 days were allowed for recovery. During the recovery period, the mice were handled, weighed, and acclimatized to the laboratory and investigators.

### Experimental procedures

#### Susceptibility to reperfusion‐induced sustained ventricular tachycardia

Conscious, unrestrained mice were studied in their home cages (standard mouse polycarbonate cage, 17 cm W × 27 cm L × 12 cm H) during the light cycle for all experiments. Cardiac output and the ECG were recorded by taping the leads to single‐stranded stainless steel wires from a miniature fluid and electric swivel (Alice King Chatham Medical Arts, Hawthorne, CA). The ECG signals were initially amplified (1000 times) with a Grass P5 11 differential preamplifier and high‐impedance probe (HIP 511GA Grass Instruments Co., Quincy, MA). The low and high pass filters were set at 0.3 Hz and 10 kHz. Cardiac output was recorded by connecting the pulsed Doppler flow probe wires to a multichannel ultrasonic flow‐dimension system with 20‐MHz high‐velocity modules (Baylor College of Medicine). The Doppler flow‐dimension system measures blood flow velocity in kilohertz of Doppler shift, which is directly proportional to absolute blood flow as determined with an electromagnetic system (Haywood et al. 1981). The temperature within the cage was monitored and maintained near the thermoneutral zone for mice of approximately 29–31°C (Swoap et al. 2004) by use of a circulating water pad under the cage and a Presto^®^ HeatDish^®^ Plus Parabolic Heater. Mice were allowed to adapt to the laboratory environment for approximately 2 h to ensure stable hemodynamic conditions.

**Figure 1.**
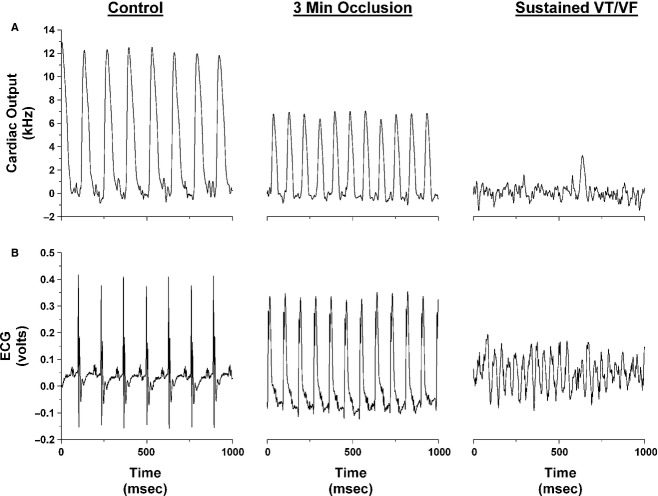
One second original recordings of cardiac output (A) and the electrocardiogram (ECG; B) before occlusion of the left main coronary artery (control), at 3 min of occlusion, and during reperfusion in an intact conscious mouse. In this mouse, reperfusion elicited sustained ventricular tachycardia (VT) within 15.35 sec.

**Figure 2.**
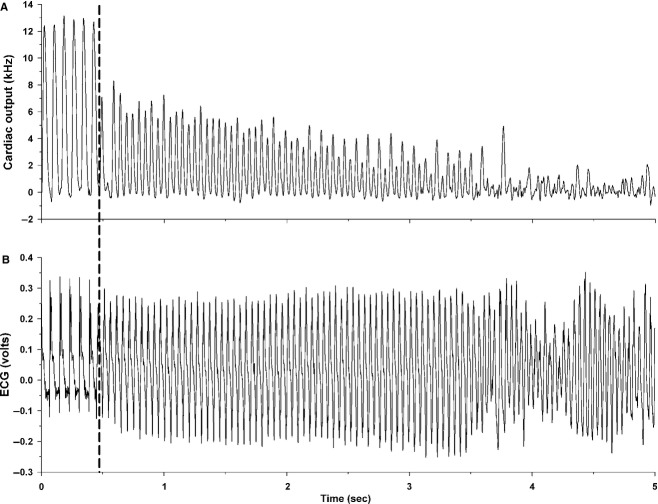
Five seconds original recordings of cardiac output (A) and the electrocardiogram (ECG; B) during the reperfusion period in an intact, conscious, male mouse. The dotted line indicates the onset of sustained ventricular tachycardia. In this mouse, reperfusion elicited sustained ventricular tachycardia within 14.65 sec.

After the stabilization period, the left main coronary artery was temporarily occluded for 3 min by use of the Prolene suture as previously described (Lujan et al. 2012a; Lujan and DiCarlo 2013). Specifically, acute coronary artery occlusion was performed by pulling up on the suture that was around the left main coronary artery and holding the occlusion for 3 min. Rapid changes in the ECG (peaked T wave followed by ST segment elevation) and reductions in cardiac output occurred within seconds of pulling on the suture, documenting coronary artery occlusion (Fig. 1) (Lujan et al. 2012a; Lujan and DiCarlo 2013). Upon release all animals sustained ventricular tachycardia (Fig. 2). Ventricular tachycardia (VT) was defined as a sustained ventricular rate (absence of P wave, wide bizarre QRS complex) >900 beats/min with a reduction in cardiac output (Curtis et al. 2013). When sustained ventricular tachycardia developed, normal sinus rhythm appeared by gently compressing the thorax and in some cases required person to mouse ventilation via a small tube over the nose. Without intervention, the sustained ventricular tachycardia progresses to ventricular fibrillation (VF). Ventricular fibrillation was defined as a ventricular rhythm without a recognizable QRS complex, in which signal morphology changed from cycle to cycle, and for which it was impossible to estimate heart rate (Curtis et al. 2013). Without intervention, the sustained ventricular tachycardia results in death. The reperfusion protocol was repeated once per week for 3 weeks. After the third protocol, the animals were sacrificed and the hearts were harvested.

#### Preparation of heart sections

Following the completion of the studies, the hearts were excised under deep anesthesia and processed as recently described (Lujan et al. 2012a; Lujan and DiCarlo 2014). The heart was quickly rinsed in 10 mmol/L Tris, 0.9% NaCl, 0.05% thimerosal in 10 mmol/L phosphate buffer, pH 7.4 (TPBS) then immersion fixed in formaldehyde/zinc fixative for 60 min, washed in TPBS (3 × 10 min), then cryoprotected overnight in 30% sucrose (prepared in half strength TPBS). The hearts were embedded in OCT compound, sliced transversely from the apex to the base (short axis) at 10‐micron thickness with the use of a cryostat. An interval of 300 microns was maintained between the ventricular sections. All sections were thaw mounted on Superfrost Plus slides and stained with Masson Trichrome.

### Data analysis

All physiological recordings were sampled at 4 kHz, and the data were expressed as means ± SE. A two‐way repeated measures analysis of variance (ANOVA) was used to determine time (weeks 1, 2, and 3) and treatment (occlusion and reperfusion) changes in cardiac output and heart rate before occlusion of the left main coronary artery, during minutes 1, 2, and 3 of occlusion and following 30 min of reperfusion. The Holm–Sidak post hoc procedure was used for post hoc pair wise comparisons. A Student's paired *t*‐test was used to determine differences in body weight between the day of the surgery and at the completion of the studies. Finally, a one‐way repeated measures ANOVA was used to determine differences in the time to VT, following reperfusion, during weeks 1, 2, and 3. A value of *P *<**0.05 was considered statistically significant.

Cardiac output was evaluated using an ultrasonic range‐gated pulsed Doppler flow meter. Blood flow (Q), in mL/min, was calculated by: 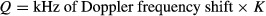
where *K* = 1.24 × *d*^2^ (*d* is the cuff diameter of the flow probe) (Haywood et al. 1981). Using this system, the Doppler shift frequency (kHz) is directly proportional to blood flow.

## Results

Figure 1 is an original recording of cardiac output and the ECG before occlusion of the left main coronary artery (control) at 3 min of occlusion and following reperfusion (15.35 sec) in an intact conscious male mouse. Coronary artery occlusion significantly reduced cardiac output. The ECG showed a peaked T wave followed by ST segment elevation. Upon release the animals experienced sustained ventricular tachycardia within seconds (week 1: 10.85 ± 3.02; week 2: 11.95 ± 1.65, and week 3: 9.96 ± 1.42). There was no difference in the time to VT between the weeks. Without intervention, the sustained ventricular tachycardia progresses to VF and results in death.

Figure 2 is an original recording of cardiac output and the ECG during reperfusion leading to sustained ventricular tachycardia in an intact, conscious, male mouse. Reperfusion elicited sustained ventricular tachycardia within 14.65 sec in this mouse. Normal sinus rhythm appeared by gently compressing the thorax and in some cases required person to mouse ventilation via a small tube over the nose. Without intervention, the sustained ventricular tachycardia progresses to VF.

**Figure 3.**
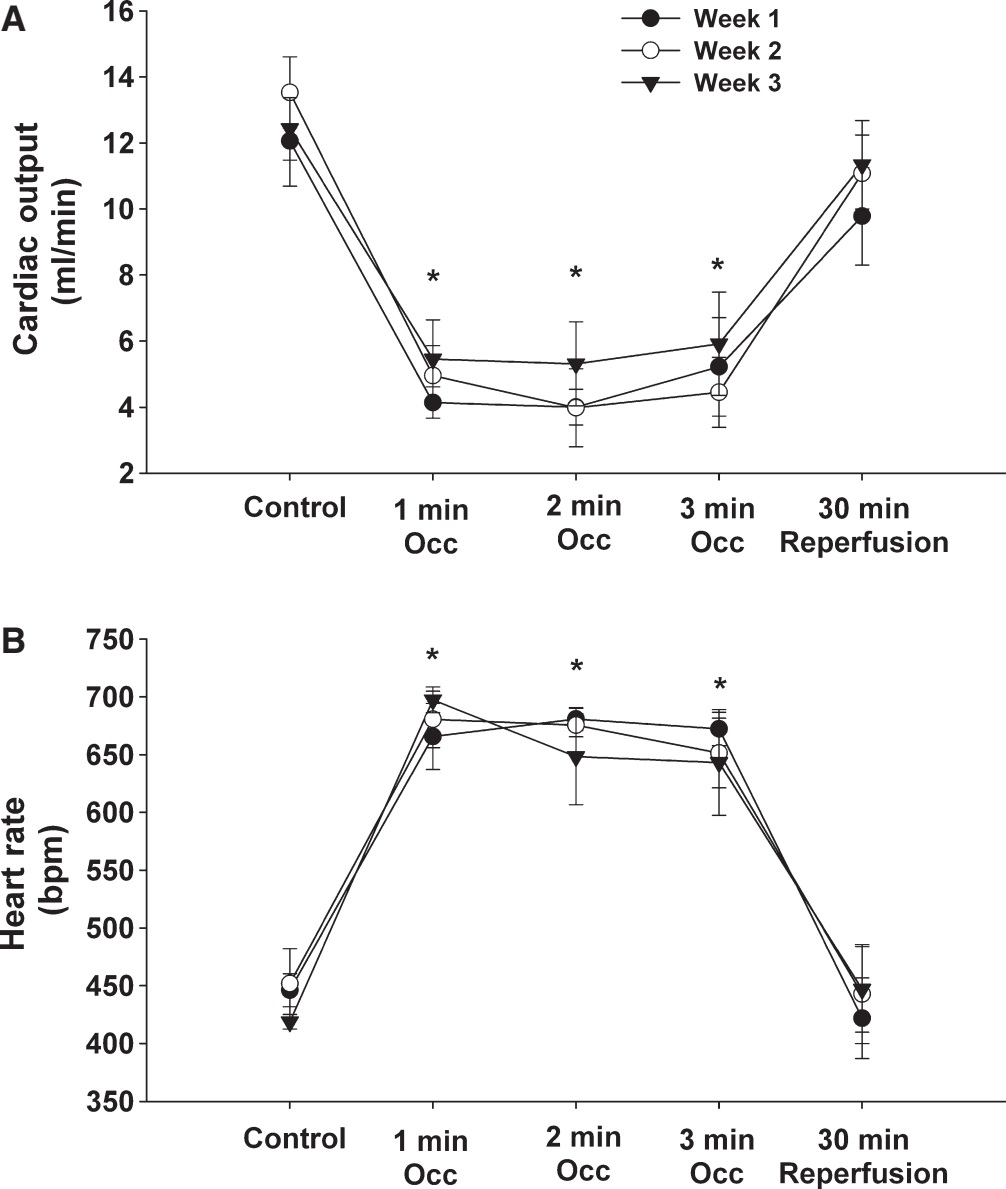
Cardiac output (A) and heart rate (B) during weeks 1, 2, and 3 before occlusion (control) of the left main coronary artery, during minutes 1, 2, and 3 of occlusion and following 30 min of reperfusion in intact conscious mice. *P* < 0.05, control versus during occlusion.

Figure 3 presents cardiac output (panel A) and heart rate (panel B) (obtained under normal sinus rhythm during weeks 1, 2, and 3) before occlusion (control) of the left main coronary artery, during minutes 1, 2, and 3 of occlusion and following 30 min of reperfusion in intact conscious mice. There was a significant treatment effect without a significant time effect or treatment by time interaction. Specifically, acute coronary artery occlusion significantly reduced cardiac output and increased heart rate. Cardiac output and heart rate returned to preocclusion values within 30 min of reperfusion suggesting that little or no tissue damage occurred during the ischemia. Finally, the response to coronary artery occlusion and reperfusion was not different during weeks 1, 2, or 3.

**Figure 4.**
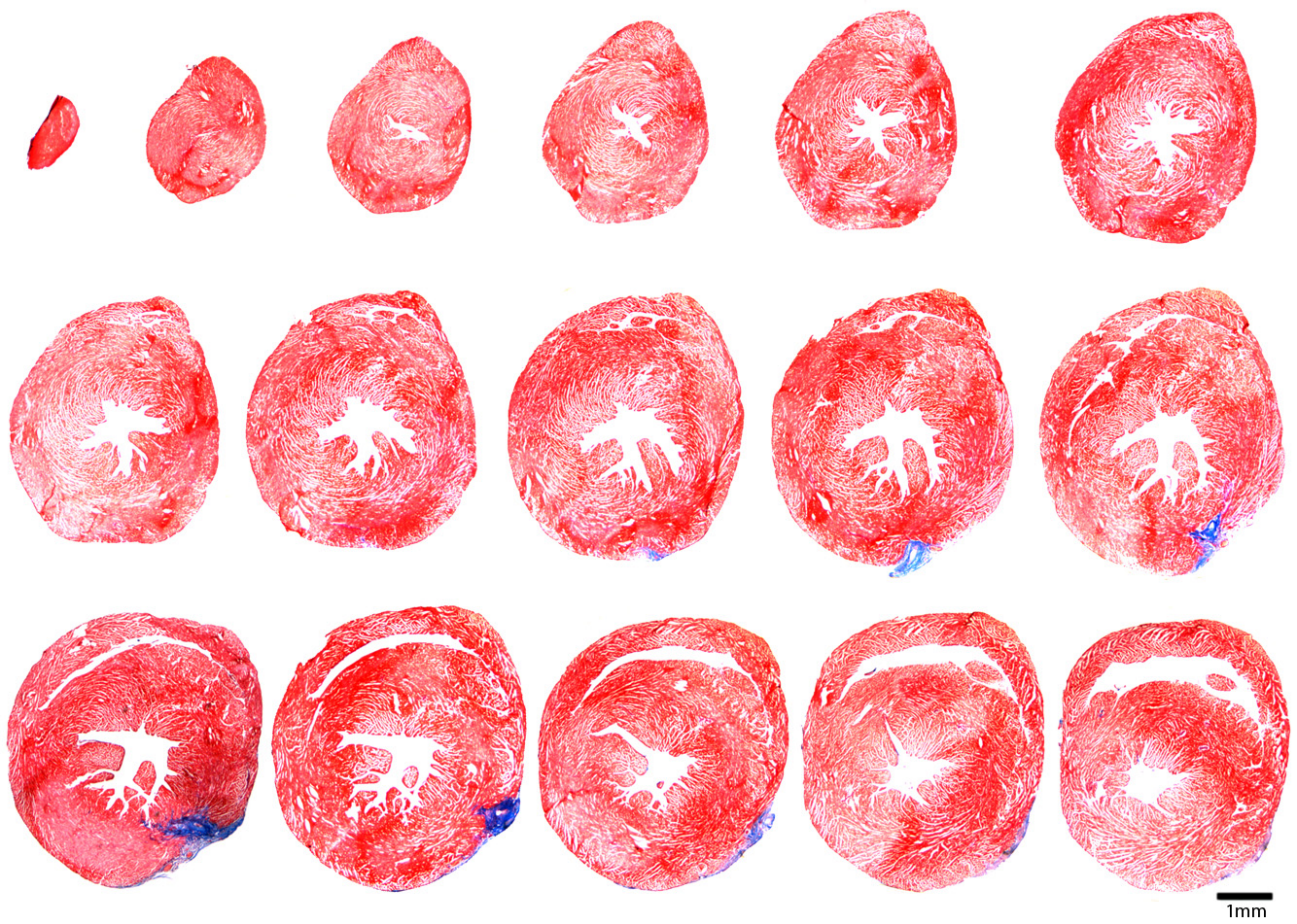
Photomicrographs of ventricular sections from one chronically instrumented mouse heart. The only collagen (i.e., blue stain, documenting tissue injury) shows placement of the coronary artery occluder. Thus, little tissue damage occurs during the ischemia; however, life threatening arrhythmias occurred during reperfusion.

Photomicrographs of ventricular sections from one chronically instrumented mouse heart are presented in Figure 4. The 10‐micron sections were taken from the apex through the base (short axis) of the left ventricle at 300‐micron intervals and processed with Masson Trichrome stain. The only collagen (i.e., blue stain, documenting tissue injury) shows placement of the coronary artery occluder. Thus, although little tissue damage occurs during the ischemia, life threatening sustained ventricular tachycardia occurred during reperfusion.

## Discussion

Acute myocardial ischemia‐induced VF accounts for approximately 50% of all sudden and unexpected cardiac deaths (Huikuri et al. 2001; Zipes et al. 2006). Reperfusion, following coronary artery occlusion, may account for some cases of sudden cardiac death (Manning and Hearse 1984; Van Wagoner and Bond 2001). In this study, for the first time, we recorded the susceptibility to sustained ventricular tachycardia, leading to VF, induced by myocardial ischemia and reperfusion in chronically instrumented, intact conscious mice. The sustained ventricular tachycardia occurred within seconds of the start of reperfusion (week 1: 10.85 ± 3.02; week 2: 11.95 ± 1.65, and week 3: 9.96 ± 1.42) which is consistent with other nonmurine models (Manning and Hearse 1984; Curtis and Hearse 1989a,b).

Although reperfusion‐induced sustained ventricular tachycardia leading to VF has been recorded in many models (Murdock et al. 1980; Balke et al. 1981; Manning and Hearse 1984; Bernier et al. 1989), reports in mice are sparse and of limited success. For example, only one study has reported successful reperfusion‐induced VF in mice (Stables and Curtis 2009) however; similar studies were unsuccessful in producing reperfusion‐induced VF (Harrison et al. 1998; Sakamoto et al. 1999; Maekawa et al. 2002). Importantly, none of these studies were conducted in intact, conscious mice.

Specifically, Stables and Curtis (2009), using the isolated Langendorff‐perfused mouse model, were the first investigators to develop a practicable model of reperfusion‐induced VF in the isolated mouse heart. Importantly, this model required perfusion with low potassium, physiological supplementation of catecholamines, and right atrial pacing at 600 beats/min. Even with these conditions, the incidence of VF was only 71% during reperfusion (Stables and Curtis 2009). Of interest is the observation that the highest incidence of VF occurred 5–10 min after reperfusion which contrast sharply with what occurs in the intact conscious mouse. In addition, the investigators used a longer duration of ischemia (30 min). Similarly, anesthetized, in vivo murine models of coronary artery occlusion were unsuccessful in eliciting reproducible sustained ventricular tachyarrhythmias (Harada et al. 1998; Harrison et al. 1998; Sakamoto et al. 1999; Lerner et al. 2000; Gehrmann et al. 2001; Maekawa et al. 2002; Mancuso et al. 2003; Lai et al. 2004). This is consistent with a recent report documenting that anesthesia and surgical trauma virtually eliminated the ability to induce sustained ventricular tachyarrhythmias in mice via programed electrical stimulation (Lujan and DiCarlo 2014).

It is also interesting to note that the incidence of reperfusion‐induced ventricular arrhythmias has a bell‐shaped relationship with the duration of preceding ischemia. Specifically, ventricular arrhythmias initially increase with increasing ischemic duration, then peaks and starts to decline with longer duration. For example, the peak incidence of ventricular arrhythmias occur after 5 min of ischemia in rats in vivo (the peak occurs after longer [15 min] ischemic duration in isolated rat hearts) (Manning and Hearse 1984). Similarly, in man, reperfusion arrhythmias typically have a very narrow time window (i.e., the peak incidence occurs after less than an hour of ischemia). As such, reperfusion arrhythmias are rare in the majority of the patients receiving thrombolytic therapy because the intervention is typically initiated a few hours after the onset of ischemia (Hearse and Bolli 1992). However, Hearse and Bolli (1992) have suggested that the development of new thrombolytic therapies that will be administered earlier, before arriving at the hospital, will increase the incidence of reperfusion‐induced lethal arrhythmias. Taken together, reperfusion‐induced lethal arrhythmias may be most important after brief periods of ischemia (e.g., relief of coronary artery vasospasm, unstable angina, exercise‐induced ischemia, and silent ischemia) (Previtali et al. 1983; Myerburg et al. 1992; Lie 1993).

As reperfusion‐induced VF is seldom reported in mice, most investigators use alternate parameters of arrhythmias (Curtis et al. 2013) including ventricular tachycardia episodes (Harrison et al. 1998; Sakamoto et al. 1999; Lerner et al. 2000; Mancuso et al. 2003; Lai et al. 2004), ventricular premature beat frequency (Harrison et al. 1998; Maekawa et al. 2002), or electrically induced arrhythmia incidence (Lerner et al. 2000; Gehrmann et al. 2001). However, these alternate parameters of arrhythmias may be of questionable clinical significance (Bourke et al. 1995; Buxton et al. 2002; Spector 2005). For example, arrhythmias induced by programed electrical stimulation do not predict the risk of VF or the effectiveness of antiarrhythmic agents (Bourke et al. 1995; Spector 2005). Furthermore, the CAST (Cardiac Arrhythmia Suppression Trial) demonstrated that antiarrhythmic drugs that prevented ventricular ectopic beats not only failed to prevent sudden cardiac death but actually increased overall mortality (CAST Investigators 1989; Akhtar et al. 1990; CAST II Investigators 1992). These data suggest that the occurrence of ventricular premature beats, bigeminy, and salvos may be of limited clinical significance and that the incidence of ventricular tachycardia leading to VF may be the most important endpoint in models of sudden cardiac death (Stables and Curtis 2009).

## Conclusion

Acute coronary artery occlusion is the leading cause of death in industrially developed countries and will be the major cause of death in the world by the year 2020 (Murray and Lopez 1997). The majority of these deaths result from tachyarrhythmias that culminate in VF (Hinkle and Thaler 1982; de Luna Bayes et al. 1989). Accordingly, models of coronary artery occlusion‐induced sustained ventricular tachycardia have the potential to be of major importance for advancing the concepts and methods that drive antiarrhythmic therapies. Importantly, to date, all in vivo murine models of coronary artery occlusion are performed in anesthetized animals with acute surgical trauma (Harada et al. 1998; Sakamoto et al. 1999; Gehrmann et al. 2001; Maekawa et al. 2002; Lai et al. 2004). Furthermore, none of these studies were successful in eliciting reproducible sustained ventricular tachyarrhythmias (Harada et al. 1998; Harrison et al. 1998; Sakamoto et al. 1999; Lerner et al. 2000; Gehrmann et al. 2001; Maekawa et al. 2002; Mancuso et al. 2003; Lai et al. 2004). In sharp contrast, in the intact, conscious mouse, the induction of sustained ventricular tachycardia is reliable. Furthermore, the procedures conducted in conscious C57BL/6J mice, a strain commonly used in transgenic studies, can be utilized in genetically modified models to enhance our understanding of single gene defects on reperfusion‐induced arrhythmias in intact, conscious animals. Finally, the coronary artery occlusion and reperfusion protocol can be initiated after the resolution of the inflammation that occurs during the initial surgical preparation.

The chronically instrumented murine model also has several labor, cost, and animal saving advantages over acute models or isolated heart preparations. Specifically, although the initial preparation and surgical instrumentation is slightly more labor intensive, once prepared, the same animal can be studied repeatedly over weeks saving preparation and instrumentation time, as well as money and animals (Fig. 3). This is consistent with the “three Rs” of animal testing first described by Russell and Burch in 1959. Specifically, investigators are encouraged to use methods that obtain comparable levels of information from fewer animals, or to obtain more information from the same number of animals. The chronic model obtains more information from fewer animals. Accordingly, the chronic model can be used in drug or other screening protocols. Another advantage of this chronic mouse model is in testing the arrhythmic consequences of genetic manipulations. As such, it is a useful addition to the range of methods already available to study arrhythmias in other settings (e.g., myocardial infarction and heart failure) in conscious mice, and would complement ex vivo isolated heart approaches to study reperfusion‐induced arrhythmias.

Despite these advantages, it is important to acknowledge that there are significant electrophysiological differences between mice and humans that could profoundly influence arrhythmic behavior (Nerbonne 2004; Kaese and Verheule 2012). Furthermore, data gathered from experiments performed at many levels, from molecules to man, will be critical for understanding reperfusion‐induced lethal arrhythmias (Lujan et al. 2012a). Accordingly, a wide range of investigations, rather than a single model is required. In this context, the conscious mouse provides an additional tool for understanding reperfusion‐induced lethal arrhythmias.

## Conflict of Interest

None declared.
